# Supplementary material for: Overlapping nuclear import and export paths unveiled by two-colour MINFLUX
Source: Nature. 2025 Mar 19;640(8059):821–7. doi: 10.1038/s41586-025-08738-0 (PMC12003200; doi:10.1038/s41586-025-08738-0)
Supplement: Supplementary file 1 — Reporting Summary [file 41586_2025_8738_MOESM1_ESM.pdf]

nature portfolio

Corresponding author(s): Siegfried Musser

Last updated by author(s): 02/03/2025

Reporting Summary

Nature Portfolio wishes to improve the reproducibility of the work that we publish. This form provides structure for consistency and transparency in reporting. For further information on Nature Portfolio policies, see our Editorial Policies and the Editorial Policy Checklist.

Statistics

For all statistical analyses, confirm that the following items are present in the figure legend, table legend, main text, or Methods section.

n/a	Confirmed	
☐	☒	The exact sample size (*n*) for each experimental group/condition, given as a discrete number and unit of measurement
☐	☒	A statement on whether measurements were taken from distinct samples or whether the same sample was measured repeatedly
☒	☐	The statistical test(s) used AND whether they are one- or two-sided<br>*Only common tests should be described solely by name; describe more complex techniques in the Methods section.*
☒	☐	A description of all covariates tested
☐	☒	A description of any assumptions or corrections, such as tests of normality and adjustment for multiple comparisons
☐	☒	A full description of the statistical parameters including central tendency (e.g. means) or other basic estimates (e.g. regression coefficient) AND variation (e.g. standard deviation) or associated estimates of uncertainty (e.g. confidence intervals)
☒	☐	For null hypothesis testing, the test statistic (e.g. *F*, *t*, *r*) with confidence intervals, effect sizes, degrees of freedom and *P* value noted<br>*Give P values as exact values whenever suitable.*
☒	☐	For Bayesian analysis, information on the choice of priors and Markov chain Monte Carlo settings
☒	☐	For hierarchical and complex designs, identification of the appropriate level for tests and full reporting of outcomes
☒	☐	Estimates of effect sizes (e.g. Cohen's *d*, Pearson's *r*), indicating how they were calculated

Our web collection on statistics for biologists contains articles on many of the points above.

Software and code

Policy information about availability of computer code

Data collection	Data collection was performed on microscopes as detailed in the manuscript using:<br>CasAO 1.0, MiCAO 1.3, LabVIEW 2015, Micro-Manger 2.0 (Zeiss 200M; astigmatism microscope for 3D localization of single molecules)<br>Abberior Imspector 16.3.13924-m2112 (MINFLUX microscope)<br>Luminosa 1.0.0.4067 (Luminosa microscope)
Data analysis	Data were curated and analyzed with custom code written in MATLAB R2018z and MATLAB 2022b. This custom code was essential to the analysis and central for extracting conclusions from the data. All MATLAB scripts used for data analysis, along with default parameters and model data, are available in the GitHub repository (https://github.com/npctat2021/MINFLUX_NPC_Tracking). A user guide for the scripts is provided in the repository's 'README' section.<br>The following commercial software packages were used for data analysis, data presentation, and simulations:<br>Imspector 16.3.15620-m2205-MINFLUX_BASE<br>ParaView 5.8.1<br>Image J (Fiji 1.52P)<br>Origin 8.5<br>Kaleidagraph 5.01<br>Microsoft Excel 16.76 (23081101)<br>The Microsoft Excel Spreadsheet used for simulating jump steps (Jump Step Histogram Simulator.xlsx) is provided in the Supplementary Information.

For manuscripts utilizing custom algorithms or software that are central to the research but not yet described in published literature, software must be made available to editors and reviewers. We strongly encourage code deposition in a community repository (e.g. GitHub). See the Nature Portfolio guidelines for submitting code & software for further information.

Data

Policy information about availability of data

All manuscripts must include a data availability statement. This statement should provide the following information, where applicable:

- Accession codes, unique identifiers, or web links for publicly available datasets
- A description of any restrictions on data availability
- For clinical datasets or third party data, please ensure that the statement adheres to our policy

The NPC scaffolds shown in Fig. 1a, Fig. 2a, Fig. 2b, and Fig. 4f are from https://www.nature.com/articles/nsmb.3244, https://doi.org/10.1126/science.add2210, https://pdb101.rcsb.org/motm/205, and https://www.sciencedirect.com/science/article/pii/S0092867421000684, respectively. All data described in the manuscript are shown in the figures and provided in Supplementary Information. Source data for the main figures and extended data figures are provided as Supplementary Information. SI All Transport contains the coordinates for all 225 Imp alpha trajectories transiting an NPC. Due to size, raw MINFLUX imaging data is not provided as part of the manuscript, but is available from the authors upon request.

Research involving human participants, their data, or biological material

Policy information about studies with human participants or human data. See also policy information about sex, gender (identity/presentation), and sexual orientation and race, ethnicity and racism.

Reporting on sex and gender	N/A
Reporting on race, ethnicity, or other socially relevant groupings	N/A
Population characteristics	N/A
Recruitment	N/A
Ethics oversight	N/A

Note that full information on the approval of the study protocol must also be provided in the manuscript.

Field-specific reporting

Please select the one below that is the best fit for your research. If you are not sure, read the appropriate sections before making your selection.

☒ Life sciences ☐ Behavioural & social sciences ☐ Ecological, evolutionary & environmental sciences

For a reference copy of the document with all sections, see nature.com/documents/nr-reporting-summary-flat.pdf

Life sciences study design

All studies must disclose on these points even when the disclosure is negative.

Sample size	No statistical methods were used to predetermine sample size. Single molecule localizations numbered in the thousands to tens of thousands and were collected for hundreds of nuclear pores, tens of cells and multiple days. The nuclear pore scaffold structures matched expectations from previous studies, indicating sufficient localizations. Transport trajectory behaviors were reproduced between two datasets, indicating reproducibility.
Data exclusions	Acceptable criteria for HMSiR localizations were CFR < 0.8 and 60 kHz > EFO > 25 kHz (dataset 1) or 100 kHz > EFO > 50 kHz (dataset 2). Acceptable criteria for trajectories were ≥ 5 localizations, CFR < 0.8 and DCR < 0.5. Data exclusion criteria are discussed in the Methods and main text.
Replication	All experimental results were successfully reproduced using different samples and measured on different days. The number of all experimental replicates are specified in the legends of all figures.
Randomization	The single molecule data collected were, by their very nature, random. This study did not allocate experimental groups, thus no randomization of data between groups was necessary.
Blinding	Blinding was not performed. Data was not allocated into different groups for comparison, and no a priori was assumed about the present observations, so blinding was unnecessary.

Reporting for specific materials, systems and methods

We require information from authors about some types of materials, experimental systems and methods used in many studies. Here, indicate whether each material, system or method listed is relevant to your study. If you are not sure if a list item applies to your research, read the appropriate section before selecting a response.

Materials & experimental systems

n/a	Involved in the study
☐	☒ Antibodies
☐	☒ Eukaryotic cell lines
☒	☐ Palaeontology and archaeology
☒	☐ Animals and other organisms
☒	☐ Clinical data
☒	☐ Dual use research of concern
☒	☐ Plants

Methods

n/a	Involved in the study
☒	☐ ChIP-seq
☒	☐ Flow cytometry
☒	☐ MRI-based neuroimaging

Antibodies

Antibodies used	The anti-GFP nanobody LaG-9 was reported previously (Fridy et al., 2014, Nat. Meth. 11:1253). This is not a commercially available nanobody but was created by the authors of the indicated paper.
Validation	The LaG-9 nanobody was validated by comparing binding to a GFP-tagged cell line relative to its untagged control. We reported this earlier (Chowdhury, Sau, & Musser. 2022 Nat. Cell Biol. 24:112).

Eukaryotic cell lines

Policy information about cell lines and Sex and Gender in Research

Cell line source(s)	U-2 OS-CRISPR-NUP96-mEGFP clone #195 (300174, CLS GmbH) and U-2 OS (300364, CLS GmbH)
Authentication	Used without authentification.
Mycoplasma contamination	Cell lines were not tested for mycoplasma contamination since they were used within one month after thawing.
Commonly misidentified lines (See ICLAC register)	None.

Plants

Seed stocks	N/A
Novel plant genotypes	N/A
Authentication	N/A
