# Supplementary material for: Overlapping nuclear import and export paths unveiled by two-colour MINFLUX
Source: Nature. 2025 Mar 19;640(8059):821–7. doi: 10.1038/s41586-025-08738-0 (PMC12003200; doi:10.1038/s41586-025-08738-0)
Supplement: Supplementary file 2 — Parameters used for NbGFP-HMSiR 3D localizations. [file 41586_2025_8738_MOESM2_ESM.docx]

**SI Table 1 | Parameters used for Nb^GFP^-HMSiR 3D localizations**

|  | Iteration^a^ | | | | | | | |
| --- | --- | --- | --- | --- | --- | --- | --- | --- |
|  | 1^st^ | 2^nd^ | 3^rd^ | 4^th^ | 5^th^ | 6^th^ | 7^th^ | 8^th^ |
| *L* size (nm) | 288 | 1440 | 288 | 288 | 151 | 151 | 75 | 75 |
| TCP | Hexagon | Zline | Square | Zline2 | Square | Zline2 | Square | Zline2 |
| Minimum number of collected photons | 40 | 400 | 100 | 30 | 40 | 25 | 50 | 25 |
| 642 nm laser power^b^ | 1x | 1x | 1x | 1x | 2x | 2x | 4x | 4x |
| Minimum TCP dwell time (ms) | 1 | 1 | 1 | 1 | 1 | 1 | 1 | 1 |
| Pattern repeat | 1 | 1 | 3 | 3 | 3 | 3 | 3 | 3 |
| CFR check |  |  |  |  | < 0.9 |  | < 0.8 |  |
| Background threshold (kHz) | 15 | 15 | 15 | 15 | 15 | 15 | 15 | 15 |

^a^Iterations 1-6 were used for initial fluorescent particle localization and the 7^th^ and 8^th^ iterations were used to continuously locate the particle until it was lost.

^b^1x = 14.2 µW, measured at the sample plane.
