# Supplementary material for: Overlapping nuclear import and export paths unveiled by two-colour MINFLUX
Source: Nature. 2025 Mar 19;640(8059):821–7. doi: 10.1038/s41586-025-08738-0 (PMC12003200; doi:10.1038/s41586-025-08738-0)
Supplement: Supplementary file 3 — NPC scaffold parameters determined from HMSiR localizations for different experiments. [file 41586_2025_8738_MOESM3_ESM.docx]

**SI Table 2 | NPC scaffold parameters determined from HMSiR localizations for different experiments^a^**

| Experiment | Method | Cells | NPCs | HMSiR Localizations | Inter-Ring Spacing (nm) | *z* Width,  σ_zw_ (nm)^b^ | Mean Radius (nm) | Radial Width,  σ_rw_ (nm)^b^ |
| --- | --- | --- | --- | --- | --- | --- | --- | --- |
| Imp β1-mEosEM  (Fig. 4c) | Astigmatism | 48 | 412 | 5594 | 50.2 | 12.4 | 51.2 | 12.1 |
| NLS-BFP-mEosEM  (Fig. 4d) | Astigmatism | 39 | 391 | 4348 | 52.1 | 13.2 | 51.4 | 12.1 |
| ‘Ran mix’ wash of Imp β1-mEosEM (Extended Data Fig. 9l) | Astigmatism | 24 | 343 | 3512 | 52.4 | 12.4 | 52.3 | 11.9 |
| ‘transport mix – high α’ wash of NLS-BFP-mEosEM (Extended Data Fig. 9p) | Astigmatism | 18 | 307 | 3780 | 52.6 | 12.1 | 50.6 | 12.6 |
| Cargo Import^c^ | Astigmatism | 20 | 257 | 3370 | 50.4 | 11.3 | 52.3 | 11.6 |
| Average/total from Astigmatism Data^d^ |  | 149 | 1710 | 20,604 | 51.5±1.1 | 12.3±0.7 | 51.6±0.7 | 12.1±0.4 |
| Imp α-JF549 tracking  (Extended Data Fig. 4a,b,c) | 3D MINFLUX  (raw) | 37 | 541 | 82,331 | 76.8±0.8 | 20.2±0.2 | 51.1±1.4 | 15.5±0.3 |
| Imp α-JF549 tracking  (Extended Data Fig. 4g,h,i)^e^ | 3D MINFLUX  (*z* scaled) | 37 | 541 | 82,331 | 51.6±0.6 | 12.8±0.1 | 51.1±1.4 | 15.5±0.3 |

^a^See Extended Data Fig. 4 for histograms and images.

^b^Gaussian width, e.g., based on histograms like those in Extended Data Fig. 4.

^c^Determined from the combined dataset reported in Table 1 of reference ^6^.

^d^Arithmetic average or totals of the five previous rows. Errors are standard deviations.

^e^The *z* scaling factor for MINFLUX data was determined from the average inter-ring spacing for the astigmatism data (51.5 nm) divided by the inter-ring spacing determined from the raw MINFLUX data (76.8 nm), i.e., *z* scaling factor = 51.5 nm/76.8 nm = 0.67. Revised parameters were then estimated from the scaled data.
