# Supplementary material for: Overlapping nuclear import and export paths unveiled by two-colour MINFLUX
Source: Nature. 2025 Mar 19;640(8059):821–7. doi: 10.1038/s41586-025-08738-0 (PMC12003200; doi:10.1038/s41586-025-08738-0)
Supplement: Supplementary file 4 — Parameters used for Imp α-JF549 3D tracking. [file 41586_2025_8738_MOESM4_ESM.docx]

**SI Table 3 | Parameters used for Imp α-JF549 3D tracking**

|  | Iteration^a^ | | | | |
| --- | --- | --- | --- | --- | --- |
|  | 1^st^ | 2^nd^ | 3^rd^ | 4^th^ | 5^th^ |
| *L* size (nm) | 252 | 1260 | 252 | 132 | 88 |
| TCP | Hexagon | Zline | Octahedron | Octahedron | Octahedron |
| Minimum number of collected photons  Dataset 1  Dataset 2 | 25  25 | 30  30 | 40  40 | 40  40 | 25  20 |
| 561 nm laser power^b^ | 1x | 1x | 1x | 2.5x | 6x |
| Minimum TCP dwell time (ms) | 0.3 | 0.3 | 0.3 | 0.3 | 0.3 |
| Pattern repeat | 1 | 1 | 1 | 1 | 1 |
| CFR check  Dataset 1  Dataset 2 |  |  | < 0.8  < 0.8 |  | < 2  < 3 |
| Background threshold (kHz)  Dataset 1  Dataset 2 | 10  10 | 10  10 | 10  10 | 20  10 | 30  45 |

^a^Iterations 1-4 were used for initial fluorescent particle localization and the 5^th^ iteration was used to continuously locate the particle until it was lost.

^b^1x = 66.7 µW, measured at the sample plane.
