# Supplementary material for: Overlapping nuclear import and export paths unveiled by two-colour MINFLUX
Source: Nature. 2025 Mar 19;640(8059):821–7. doi: 10.1038/s41586-025-08738-0 (PMC12003200; doi:10.1038/s41586-025-08738-0)
Supplement: Supplementary file 5 — Summary of 3D MINFLUX tracking results for Imp α-JF549. [file 41586_2025_8738_MOESM5_ESM.docx]

**SI Table 4 | Summary of 3D MINFLUX tracking results for Imp α-JF549**

| Total number of cells examined | 37 |
| --- | --- |
| Total number of NPCs identified | 541 |
| Total number of tracks identified with ≥ 5 localizations | 12,384 |
| Number of tracks entering a 400 nm cube centered on an NPC | 2,678 |
| Median track length (number of localizations/track within a 400 nm NPC-centered cube; minimum 5 localizations/track) | 18 |
| Mean time between successive localizations | 1.6 ms |
| Minimum time between successive localizations | 0.52 ms or  0.6 ms^a^ |
| Number of tracks that were caught transiting a pore (one or more points ≤ 25 nm from an NPC midplane) | 225 |
| Number of Import tracks (crossed from *z* = +25 nm to *z* = -25 nm) | 32 |
| Number of Export tracks (crossed from *z* = -25 nm to *z* = +25 nm) | 23 |
| Number of Abortive Import tracks (> 25 nm to < 25 nm to > 25 nm) | 45 |
| Number of Abortive Export tracks (< -25 nm to > -25 nm to < -25 nm) | 51 |
| Number of Undecided tracks (beginning or end point ≤ 25 nm from an NPC midplane) | 74 |
| Median number of localizations per Import track | 29 |
| Median time to cross the NPC barrier during Import (*z* = +25 nm to *z* = -25 nm) | 14 ms |
| Median number of localizations within the NPC barrier per Import event (*z* = +25 nm to *z* = -25 nm) | 7 |
| Median number of localizations per Export Track | 22 |
| Median time to cross the NPC barrier during Export (*z* = -25 nm to *z* = +25 nm) | 8.7 ms |
| Median number of localizations within the NPC barrier per Export event (*z* = +25 nm to *z* = -25 nm) | 4 |

^a^For dataset 1 and dataset 2, respectively.
